# Maladaptive Daydreaming: Epidemiological Data on a Newly Identified Syndrome

**DOI:** 10.3389/fpsyt.2022.871041

**Published:** 2022-04-27

**Authors:** Nirit Soffer-Dudek, Nitzan Theodor-Katz

**Affiliations:** Consciousness and Psychopathology Laboratory, Department of Psychology, Ben-Gurion University of the Negev, Beer-Sheva, Israel

**Keywords:** Maladaptive Daydreaming, daydreaming, fantasy, absorption, prevalence, epidemiology, norms, psychopathology

## Abstract

**Background:**

Maladaptive Daydreaming (MD) is a recently identified psychological disorder, characterized by excessively and addictively engaging in vivid, narrative, intensely emotional fantasy activity, at times with the aid of music and/or repetitive movements, causing distress and functional impairment. Over 100,000 self-diagnosed individuals are active online and thousands of them have been researched; yet there are no studies using clinical interviews on large, systematic general (non-MD) samples, to assess the estimated prevalence of this suggested disorder, and establish norms for its main psychometric tool.

**Methods:**

Four independent Israeli samples (three student samples, and one sample representing the general Jewish-Israeli population; total *N* = 1,023) self-reported MD. In two samples, those exceeding the cutoff score for suspected MD were invited for a structured clinical interview.

**Results:**

The skewness of most items of the 16-item Maladaptive Daydreaming Scale (MDS-16) supports the notion of MD as a binary construct rather than a normally distributed trait. In the community sample, 4.2% exceeded the cutoff for suspected MD. Rates were higher when focusing on the young adult age group or student samples (5.5–8.5%), suggesting a likely age effect. Following clinical interviews, only 60% of interviewed respondents met criteria for diagnosis, suggesting a true point-prevalence of 2.5% in the Israeli-Jewish population.

**Conclusions:**

This is the first systematic clinical evaluation of the prevalence of MD. In an Israeli sample, a point-prevalence of 2.5% was found, like several other internalizing psychiatric syndromes. This result, along with the Non-normal nature of item distribution, both support the validity of MD as a psychological disorder, which should be considered as a potential addition to future psychiatric diagnostic manuals.

## Introduction

Maladaptive Daydreaming (MD) is a proposed mental disorder characterized by excessive, compulsive immersion in vivid and complex fantastical daydreamed plots, generating intense emotional involvement, often accompanied by stereotypical movements (1–3). This addictive absorption in daydreaming becomes maladaptive as it consumes many hours a day, generates shame or guilt, hinders achievement of short- and long-term goals or tasks, and overall causes clinically significant distress and/or interferes with functioning in social or occupational realms (2, 4–7). Maladaptive Daydreamers (MDers) report a strong urge to daydream whenever they can and annoyance whenever they cannot, and, repeated unsuccessful efforts to control, cut back, or stop daydreaming, like other behavioral addictions (4, 7). Negative emotions follow their daily daydreaming activity (8, 9). However, mental health practitioners are often disparaging of their problem, resulting in suboptimal treatment and heightened loneliness and distress (1).

Importantly, different types of mental states in which one does not focus on the present may be associated with psychological difficulties, such as rumination, worry, thinking about the past or future, or mind-wandering (10, 11). However, MD is essentially different than normal daydreaming and mind-wandering, defined quite broadly as internally generated, or off-task, thought (12). Widespread daydreaming or mind-wandering is often a spontaneous divergence from a present task in favor of past or future recollections (such as activating a memory or thinking about one's to-do list for the rest of the day). Self-report mind-wandering items span inattention and concentration difficulties, a scattered line of thought, making mistakes due to automatic behavior, and obliviousness to surroundings (13). On the contrary, MD is characterized by inventing rich, fantastical plots and stories with a dynamic emotional range, that are often unrealistic and distant from the daydreamer's actual life. Moreover, they are usually continuously evolving over long periods of time, like a soap opera, which is very different than the somewhat random, fickle contents of common mind-wandering. In MD, individuals feel compelled to continue their fantasy, like many people would feel about watching their favorite show on TV, and many report that they initiate the MD episode with awareness and intent. Notwithstanding, most MDers will meet criteria for a diagnosis of an attention deficit, but they explain that it stems from their core problem of addiction to daydreaming (14). A study presenting a nosological differentiation spanning several constructs concluded that mind-wandering shared the least similarity with MD (5). These authors also cited a case of a woman who reported that previously prescribed Methylphenidate (Ritalin) improved her academic performance but seemed to increase and worsen her daydreaming addiction. Moreover, an experience-sampling study on MDers found that following nights of poor sleep, they had elevated mind-wandering on the next day, but not elevated MD, which was independent of sleep quality (15). These different pieces of evidence together suggest that MD is distinct from general mind-wandering and inattention.

Thousands of MDers are active online, sharing their experiences and supporting each other, as evident in ever-growing Facebook groups, blogs, and other cyber-forums. For example, the Reddit MD community (subreddit “r/MaladaptiveDreaming”) comprises 72.8 k members (retrieved Feb 6th, 2022). Significant public interest in the term is also evident by dedicated articles in numerous media outlets (including US News & World Report, CNN, Fox news, the Guardian, and the New York Times, to name a few). A Google search for the term Maladaptive Daydreaming produces about 659,000 results (retrieved Feb 6th, 2022). Public interest together with overwhelming email outreach to researchers by self-diagnosed MDers, have spawned continuing scientific interest and research on MD. In most of these emails, MDers express their gratitude for the existence of the term and their willingness to volunteer for research on the subject to help promote the budding field. Thus, perhaps not surprisingly, most of the published studies on the topic have relied on MDers as participants. These studies suggest that MDers are very high in psychopathological comorbidities and dysfunction levels, pointing to quite a clinical population. For example, a study conducting psychiatric structured clinical interviews on MDers found that all of them met criteria for at least one current diagnosis according to the Diagnostic and Statistical Manual of Mental Disorders–the DSM-5, (16) and almost all of them met criteria for more than one, many with multiple DSM comorbidities, mostly on the spectrum of anxiety, depression, and obsessive-compulsive disorder (OCD) and related disorders, especially skin-picking (14). In a different study, almost half of the MD sample were unemployed and over a quarter of the sample reported that they had attempted suicide at least once (9). MDers also exhibit difficulties in emotion regulation and attachment styles, (17) and are highly dissociative (18). In a sample of 510 MDers, over half of them exceeded the clinical cutoff for suspected OCD (19). Shared mechanisms between MD and OCD seemed to be intrusions and lack of cognitive control, high dissociation and a tendency for absorption, and altered embodiment experience (19).

Because research on MD is very much influenced by the strong motivation of MDers to facilitate knowledge on their condition, most existing research is based on MD samples, or on mixed samples recruited both from MD forums and from other sources. For this reason, we do not yet know the *prevalence* of MD in the general population, despite there being suggested diagnostic criteria and a structured clinical interview (3). Assessing prevalence is one of the important steps needed for eventual formal recognition of MD in psychiatric diagnostic manuals. Our clinical experience suggests that MDers have an ability for highly immersive daydreaming not shared by most people, and thus most people also do not become addicted to their daydreaming, suggesting that MD is a distinct clinical condition and not a commonplace or normal experience. On the other hand, MD does not seem to be especially rare, compared to other types of mental health diagnoses. This may be inferred by the overwhelming numbers of individuals continuously joining MD groups and approaching us directly.

Thus, the current study set out to assess norms and prevalence of MD in Israel, relying on independent (non-MD) samples. We utilized four such samples, one representing the general Jewish native-Israeli population and three additional first-year psychology undergraduate student samples. We aimed to descriptively explore the item and mean score distribution on the Maladaptive daydreaming scale (MDS), (20) the validated self-report measure used in the literature to assess MD. We also wished to look at the percentage of individuals scoring over the clinical cutoff which suggests suspected clinical-level MD, in the full sample as well as in specific groups determined by age and gender. Finally, and most importantly, in two of the student samples we conducted structured clinical interviews to those who scored over the self-reported clinical cutoff, to establish true epidemiological diagnostic rates rather than merely suspected MD.

Although this study relied on descriptive and exploratory statistics alone, we did have four specific hypotheses:

1. From our clinical and prior research experience, we observed that some individuals mark high scores on the self-report measure of MD, but when interviewed, describe worries, ruminations, obsessions, or general distractions/random mind-wandering, rather than fanciful, narrative, and creative daydreamt scenes. Lacking that special ability for immersive daydreaming may cause misunderstanding of items, especially if one is overwhelmed by non-MD anxieties or intrusive images. Our central aim in the clinical interviews was to shed light on exactly what respondents mean when they refer to their “daydreaming”, to exclude false positives from counting as MDers. Thus, a central hypothesis was that *the percentage of individuals diagnosed as having MD following a clinical interview will be lower than the percentage of respondents scoring over the cutoff based on self-report*. However, we did not know how much lower: what is the true point-prevalence of MD?
2. We hypothesized that in independently recruited samples (i.e., not from MD forums) spanning hundreds of individuals, *we will be able to identify individuals with current clinical-level MD*, meaning that MD point-prevalence will be comparable to several other psychiatric conditions that are not especially rare.
3. From our prior research experience, MD samples are over-represented by females as opposed to males [e.g., (9)], and by younger individuals as opposed to older ones, although, this may be an artifact of the extent to which different generations are active on the internet. Thus, we tentatively expected *prevalence rates to be higher in women and in younger adults*.
4. In addition to assessing prevalence, we aimed to establish norms for the MDS-16 based on general samples. Most people do not seem to have the ability for highly immersive daydreaming nor do they become addicted to it, thus most MDS items should be *significantly skewed and Non-normally distributed*. We aimed to identify which items are skewed, as they have the potential to be the most discerning between non-MD and MD individuals and could be used as screener items.

## Methods

### Participants and Procedure

The present study utilized several samples collected as part of a larger line of research, all of which were systematic samples that included the MDS. Four samples (A-D) are included in this study, but only in two were we able to perform interviews following the self-report phase: Sample A used an online survey platform, in which there is built-in anonymity, meaning that the researchers cannot reach out to certain participants to invite them for an interview; Sample B relied on older data from a previous study, so again interviews were not done; Conversely, samples C and D were collected with the current investigation in mind, and thus interviews were conducted on consenting screened individuals. In all four studies, participants provided informed consent beforehand in digital form and completed online self-report questionnaires. All four studies received an institutional ethics approval from the authors' university beforehand, in accordance with the latest version of the Declaration of Helsinki. The three student samples (B-D), all comprising first-year undergraduates, were recruited in different years. Sample sizes in all studies were determined based on our wish to conduct factor analyses on scales outside the scope of the current study, leading us to aim for at least 300 participants in each sample. In sample C, we did not reach this aim because of Covid-19-related setbacks, which is why we recruited additional participants the following year with the same procedure (sample D), together forming a large enough joint sample for our needs. However, in the present study we thought it would be more accurate to leave them separate, as the ever-changing Covid-19 situation may have caused differences between consecutive years.

#### Sample A

Adult Jewish native Israelis aged 18–65 were sampled (stratified by age and gender) for a study labeled “emotions, daydreaming, and sensory experiences” through an Israeli online survey platform called “the Sample Panel Project” (www.midgampanel.com), in August 2020. This survey platform has over 90,000 panelists who receive invitations for participation in studies in exchange for monetary reimbursement. The platform has built-in measures for ensuring the quality and integrity of the data, including a comprehension validity item quite early on. We requested about 400 respondents and ended up with data from *N* = 437 respondents who passed these tests. They answered a battery of questionnaires on daydreaming, dissociation, embodiment, psychopathology and dreaming patterns, most of which are out of the scope of the current study. Six respondents were excluded because they failed our additional comprehension control item, presented toward the end of the battery. We removed an additional 48 respondents who answered the battery in a very short time, questioning the validity of their responses. We were left with 383 community respondents (56.4% females; age *M* = 40.4, *SD* = 13.5, range 19–65).

#### Sample B

For previous research, (21) 314 students enrolled for a study on “Dissociation, attention, risk, and resilience”, completing questionnaires on psychopathology, sleep and dreaming, attention, and daydreaming, for either course credit or monetary reimbursement, from January to April 2017. Seven participants were excluded because most of their data were missing, and an additional four because of the short time it took them to complete questionnaires. Finally, two had missing MD data and thus were excluded from the present investigation, resulting in *N* = 301 respondents (74.1% females; age *M* = 23.5, *SD* = 1.4, range 18–28). MD data from this sample were never used in any previous publication.

#### Sample C

Undergraduate students (*N* = 212) enrolled for a study labeled identically to Sample A, with the same questionnaires, in exchange for course credit, from April to October 2020. All completed questionnaires within a reasonable time frame and did not have significant missing data (79.6% females; age *M* = 23.8, *SD* = 1.9, range 18–38). As will be detailed later on, 17 of them were screened and invited for an interview, and 10 agreed.

#### Sample D

Undergraduate students, initially 133, enrolled for a study identical to Samples A and C, in exchange for course credit, from April to May 2021.^1^ Two participants were excluded because most of their data were missing, and an additional four because of the short time it took them to complete questionnaires, resulting in *N* = 127 respondents (80.3% females; age *M* = 24.0, *SD* = 2.8, range 20–44). As will be detailed later on, 7 of them were screened and invited for an interview, and 5 agreed.

### Measures

In addition to demographic variables (gender and age) we assessed MD using the *Maladaptive Daydreaming Scale* (MDS-16) (14, 20). This 16-item questionnaire is the main self-report measure used in research on MD. It includes four factors: the strong, addictive urge to engage in daydreaming (Yearning); daydreaming impairing functioning and interfering with long-term life goals or daily chores (Impairment); physical movement associated with daydreaming such as accompanying facial expressions, mouthing the words, rocking, or pacing (Kinesthesia); and music as a facilitator of the daydreaming (Music) (7). The scale is reliable (3, 7, 20, 22) and has been validated in Hebrew (23). Its 11-point scale ranges from 0% (e.g., never, no distress at all) to 100% (e.g., extremely frequent, extreme distress). In the present study, Cronbach's alpha was 0.92, 0.92, 0.93, and 0.90 for samples A, B, C, and D, respectively. Scores over 40 suggest suspected clinical-level MD [(3) and see corrigendum].^2^

For the clinical interviews, we used the *Structured Clinical Interview for Maladaptive Daydreaming* (SCIMD) (3). This assessment tool was designed based on the proposed diagnostic criteria for MD and was carried out in a structured interview format based on the SCID (24) for DSM-5. The second author, a licensed clinical social worker, conducted all the diagnostic interviews online, after being trained in the past by one of the developers of the SCIMD, a senior clinical psychologist. She had plenty of experience with this interview from a previous study. The SCIMD consists of a ten-question probe for inclusion criteria and one probe for an exclusion criterion indicating that the symptoms cannot be better explained by a physiological condition or another psychopathology (e.g., drug addiction, psychotic disorders, bipolar disorder, OCD, dissociative identity disorder, or medication-induced symptoms). According to the SCIMD, psychological distress with no clear functional impairment is categorized as mild; one affected area of functioning is categorized as moderate; and more than one is categorized as severe. The interview is aimed to get a clear sense of what participants mean when they refer to their “daydreaming”, as some people call their intrusive obsessions, ruminations, or general mind-wandering or distractions–“daydreaming”. We explored the extent to which interviewees report experiencing immersive vivid, narrative, fantastical imagined storylines, scenarios, and dialogues. Moreover, some people do have the ability for immersive daydreaming, but with no significant distress or functional impairment (25). They do not meet criteria for a diagnosis of MD and were classified as negative.

### Analytic Approach

We examined: (1) descriptive data for each item and for the average MDS-16 score; (2) prevalence according to the self-report cutoff score, dissected according to age and gender; and (3) true prevalence rates, according to the diagnostic interview, in samples C and D, compared to the ones extracted from self-report data alone. Specifically, in all samples we examined what percentage of respondents were above the cutoff score for suspected MD based on self-report alone, but in samples C and D, where we were able to conduct interviews, we also examined what percentage of interviewees would eventually be diagnosed as positive for MD. Finally, we aimed to combine these pieces of information to estimate the true point-prevalence in the general Jewish-Israeli population. We did so by inferring that the true positive percentage found in samples C and D, could be generalized to samples A and B as well.

## Results

### Self-Report Data

Table 1 presents item-level descriptive statistics for each of the 16 items of the MDS, across the four samples. As can be seen in the bottom row of the table, mean scores ranged from a lower 10.77 in the community sample, to a higher 13.30, 13.57 and 14.93 in the student samples. The range of mean scores, however, was broader in the community sample (0–81.25) compared to the student samples, suggesting that the large community sample may have included some cases that were more severe than those observed in the student samples. The student samples also showed different ranges (0–57.50, 0–59.38, 0–73.75), perhaps due to their different sizes (with the larger sample including more severe cases).

**Table 1:** Item-level and mean-level descriptive statistics for the MDS-16 across samples A-D.

	**A**. ***N*** **=** **383 community**			**B**. ***N*** **=** **301 students**			**C**. ***N*** **=** **212 students**			**D**. ***N*** **=** **127 students**		
	**M** **[SD]**	**Min** **Max**	**SK** **KU**	**M** **[SD]**	**Min Max**	**SK** **KU**	**M** **[SD]**	**Min** **Max**	**SK** **KU**	**M** **[SD]**	**Min Max**	**SK** **KU**
1	19.40 [27.25]	0 100	1.47 1.15	27.81 [27.48]	0 100	0.80 −0.53	25.71 [30.76]	0 100	0.98 −0.35	27.01 [31.28]	0 100	1.05 −0.10
2	15.35 [24.33]	0 100	1.70 2.08	22.62 [23.33]	0 100	0.91 −0.12	20.47 [26.71]	0 100	1.17 0.12	23.54 [27.85]	0 100	0.97 −0.39
3	10.65 [19.45]	0 100	2.27 4.89	20.66 [25.56]	0 100	1.25 0.59	17.92 [25.93]	0 100	1.45 1.04	17.09 [24.07]	0 100	1.53 1.64
4	7.99 [16.65]	0 100	2.77 8.50	10.17 [18.65]	0 100	2.38 5.74	9.34 [17.88]	0 100	2.31 5.27	6.77 [14.58]	0 80	3.05 10.36
5	8.72 [16.62]	0 90	2.46 6.47	11.96 [17.88]	0 90	1.97 3.97	11.99 [18.28]	0 80	1.58 1.64	11.26 [19.27]	0 90	2.31 5.31
6	6.53 [14.46]	0 80	2.74 7.52	6.64 [14.87]	0 100	3.30 12.97	9.76 [19.33]	0 90	2.19 4.01	8.66 [16.68]	0 90	2.63 7.65
7	13.00 [21.79]	0 100	2.05 3.90	19.24 [23.18]	0 100	1.44 1.45	14.58 [23.43]	0 100	1.83 2.52	13.23 [19.39]	0 90	1.89 3.36
8	9.66 [19.50]	0 100	2.39 5.60	9.47 [16.68]	0 80	2.19 4.42	9.39 [19.01]	0 80	2.41 5.14	7.87 [14.89]	0 90	2.69 8.83
9	10.63 [20.77]	0 100	2.42 5.64	14.55 [21.56]	0 100	2.00 3.73	12.36 [19.40]	0 90	1.83 2.74	12.20 [18.51]	0 90	1.86 3.27
10	8.75 [16.59]	0 90	2.36 5.64	10.10 [17.35]	0 100	2.41 6.21	7.77 [15.50]	0 80	2.41 5.91	9.29 [16.24]	0 80	2.14 4.40
11	10.91 [21.06]	0 100	2.33 4.99	12.99 [20.65]	0 100	1.95 3.28	10.90 [18.91]	0 90	2.11 4.10	8.90 [16.05]	0 90	2.70 8.81
12	11.93 [21.78]	0 100	2.23 4.72	10.40 [16.61]	0 90	2.02 4.16	8.71 [16.62]	0 90	2.45 6.22	9.06 [17.06]	0 90	2.40 6.05
13	4.88 [14.63]	0 100	4.21 20.03	8.70 [17.64]	0 100	2.59 6.69	6.68 [15.53]	0 80	2.71 7.13	6.38 [16.22]	0 80	2.89 8.00
14	5.80 [15.31]	0 100	3.58 14.22	7.48 [16.58]	0 90	3.02 9.89	8.67 [18.92]	0 100	2.75 7.77	5.98 [15.03]	0 90	3.99 17.75
15	19.74 [27.36]	0 100	1.54 1.47	33.85 [28.67]	0 100	0.68 −0.56	30.09 [30.72]	0 100	0.69 −0.77	31.10 [28.07]	0 90	0.52 −1.05
16	8.30 [17.20]	0 100	2.56 6.60	12.23 [19.55]	0 90	1.77 2.44	13.22 [22.32]	0 90	1.79 2.25	14.49 [22.67]	0 90	1.72 2.09
M	10.77 [13.69]	0 81.25	1.74 3.36	14.93 [14.01]	0 73.75	1.39 1.81	13.57 [15.19]	0 59.38	1.20 0.64	13.30 [12.94]	0 57.50	1.27 1.59

*M, Mean; SD, Standard Deviation; SK, Skewness; KU, Kurtosis.*

*Rows represent items of the MDS-16. For each item, the parameters represented are mean and standard deviation (left), minimum and maximum (middle), and skewness and kurtosis (right). The bottom row depicts descriptive statistics for respondents' mean MDS-16 scores*.

In terms of Non-normality, skewness and kurtosis parameters across samples suggest that items 4–14 and 16 may better represent MD as a potentially binary construct, i.e., may better differentiate normal from abnormal daydreaming (for these items, skewness and kurtosis are both larger than 1 in all samples). Conversely, items 1–3 and 15 seem to tap onto a more continuous trait.

Further support for MD as a binary construct comes from Table 2, which presents the distribution, in each sample, of mean MDS scores across tenth percentiles of possible scores (i.e., 0–10, 10–20, etc., up to 90–100). As can be seen in the table, although possible scores may range from 0 to 100, in all samples, over 70% of the sample scored lower than 20, over 80% of the sample scored lower than 30, and over 90% of the sample scored lower than 40 (the recommended cutoff score for suspected MD).

**Table 2:** Distribution of mean MDS-16 scores across samples A-D (in percentages of respondents), and in the bottom row, distribution of suspected MD across samples A-D (in percentages), according to a cutoff of 40 on the MDS-16.

	**A**. ***N*** **=** **383**		**B**. ***N*** **=** **301**		**C**. ***N*** **=** **212**^***a***^		**D**. ***N** **=** **127***	
	***females: n = 216***	***m = 167***	***f = 223***	***m = 78***	***f = 168***	***m = 43***	***f = 102***	***m = 25***
0–9.99	64.2		45.5		51.9		46.5	
	[64.2]		[45.5]		[51.9]		[46.5]	
	64.4 [64.4]	64.1 [64.1]	46.6 [46.6]	42.3 [42.3]	52.4 [52.4]	48.8 [48.8]	47.1 [47.1]	44.0 [44.0]
10–19.99	15.2		27.6		19.3		27.5	
	[79.4]		[73.1]		[71.2]		[74.0]	
	15.2 [79.6]	14.9 [79.0]	26.5 [73.1]	30.8 [73.1]	20.2 [72.6]	16.3 [65.1]	28.4 [75.5]	24.0 [68.0]
20–29.99	10.4		12.9		14.2		15.0	
	[89.8]		[86.0]		[85.4]		[89.0]	
	11.6 [91.2]	9.0 [88.0]	12.6 [85.7]	14.1 [87.2]	13.7 [86.3]	16.3 [81.4]	13.7 [89.2]	20.0 [88.0]
30–39.99	6.0		6.4		6.6		5.5	
	[95.8]		[92.4]		[92.0]		[94.5]	
	4.2 [95.4]	8.4 [96.4]	7.6 [93.3]	2.5 [89.7]	5.4 [91.7]	11.6 [93.0]	4.9 [94.1]	8.0 [96.0]
40–49.99	2.1		4.9		3.8		3.1	
	[97.9]		[97.3]		[95.8]		[97.6]	
	1.8 [97.2]	2.4 [98.8]	4.0 [97.3]	7.7 [97.4]	3.5 [95.2]	4.7 [97.7]	3.9 [98.0]	0.0 [96.0]
50–59.99	1.3		1.7		4.2		2.4	
	[99.2]		[99.0]		[100]		[100]	
	1.9 [99.1]	0.6 [99.4]	1.4 [98.7]	2.6 [100]	4.8 [100]	2.3 [100]	2.0 [100]	4.0 [100]
60–69.99	0.5		0.7		0.0		0.0	
	[99.7]		[99.7]		[100]		[100]	
	0.9 [100]	0.0 [99.4]	0.9 [99.6]	0.0 [100]	0.0 [100]	0.0 [100]	0.0 [100]	0.0 [100]
70–79.99	0.0		0.3		0.0		0.0	
	[99.7]		[100]		[100]		[100]	
	0.0 [100]	0.0 [99.4]	0.4 [100]	0.0 [100]	0.0 [100]	0.0 [100]	0.0 [100]	0.0 [100]
80–89.99	0.3		0.0		0.0		0.0	
	[100]		[100]		[100]		[100]	
	0.0 [100]	0.6 [100]	0.0 [100]	0.0 [100]	0.0 [100]	0.0 [100]	0.0 [100]	0.0 [100]
90–100	0.0		0.0		0.0		0.0	
	[100]		[100]		[100]		[100]	
	0.0 [100]	0.0 [100]	0.0 [100]	0.0 [100]	0.0 [100]	0.0 [100]	0.0 [100]	0.0 [100]
% with mean score ≥ 40	4.2%		7.6%		8.0%		5.5%	
	(16/383)		(23/301)		(17/212)		(7/127)	
	4.6% (10/216)	3.6% (6/167)	6.7% (15/223)	10.3% (8/78)	8.3% (14/168)	7.0% (3/43)	5.9% (6/102)	4.0% (1/25)

*Each cell depicts the percentage of people in the sample (column) whose mean MDS-16 score falls within the range specified in that row (or reaching the cutoff of 40 for suspected clinical-level MD, for the bottom row). In brackets are the cumulative percentages, i.e., how many people in the sample have that score or lower (for the bottom row, brackets indicate the raw number of people reaching 40 out of the raw number of people in sample). In addition to general percentages in the samples, the table also shows percentages among females (f) and males (m).*

a *One participant in this sample identified themselves as “other” in terms of gender, and thus they are counted only in the top rows but not in the gender-grouped rows*.

At the bottom of Table 2 we present percentages when using the clinical cutoff score of 40 for suspected MD. Estimated prevalence rates range from 4.2% (community sample), to 5.5, 7.6 and 8.0% (student samples). There did not seem to be any clear-cut gender differences in a certain direction.

Next, we wished to explore age differences. These could be explored only in the community sample, as student samples comprised respondents who were nearly all in the 18–30 age group. Table 3 presents the suspected prevalence, according to the cutoff of 40, across different age groups of the community sample. As evident from the table, younger participants seem to have higher suspected MD rates compared to older ones.

**Table 3:** Distribution of suspected MD (MDS-16 score over 40) according to age group, in Sample A, i.e., the community sample (*N* = 383) (in percentages).

	**A**. ***N*** **=** **383**	
	***f = 216***	***m = 167***
Age 18–30	8.5%	
	(10/117)	
	9.8% (6/61)	7.1% (4/56)
Age 31–40	1.3%	
	(1/79)	
	1.8% (1/55)	0.0% (0/24)
Age 41–50	3.7%	
	(3/82)	
	2.6% (1/38)	4.5% (2/44)
Age 51–60	2.4%	
	(2/82)	
	4.4% (2/45)	0.0% (0/37)
Age 61–70	0.0%	
	(0/23)	
	0.0% (0/17)	0.0% (0/6)

*Each cell depicts the percentage of people in the sample reaching the cutoff of 40 for suspected clinical-level MD, according to their age group (specified in the rows). Below the percentage, in brackets, the raw number of people is indicated (out of the raw number of people in sample). In addition to general percentages in the samples, the table also shows percentages among females and males*.

### Diagnostic Interviews

Table 4 shows results from the structured clinical interviews conducted on those who had scores of over 40 from samples C and D, who consented to interview. As can be seen in the table, in each of the samples, 60% of those interviewed were eventually diagnosed as positive for MD (6 out of 10 in Sample C, and 3 out of 5 in sample D). The remaining 40% either had immersive daydreaming that was not pathological/impairing, or highly intrusive and distressing rumination or mind-wandering. One additional participant had past (recovered) MD (which could be counted if we were to calculate lifetime prevalence; however, our screening focused on current symptoms).

**Table 4:** Results of diagnostic interviews for those exceeding the suspected cutoff for MD (MDS-16 score over 40) in Samples C and D.

**Sample**	**Interviewee #**	**MDS-16**	**Sex**	**MD diagnosis**	**Comments**
**C**. ***N*** **=** **212**	1	40.63	f	No	Immersive Daydreaming, no impairment/distress
	2	42.50	f	–	*(Did not interview)*
	3	43.13	f	–	*(Did not interview)*
	4	43.75	f	–	*(Did not interview)*
	5	44.38	m	Yes	MD, Severe
	6	45.00	f	–	*(Did not interview)*
	7	46.25	m	Yes	MD, Severe
	8	49.38	f	Yes	MD, Severe
	9	51.88	f	–	*(Did not interview)*
	10	52.50	f	No	Immersive Daydreaming, no impairment/distress
	11	52.50	f	Yes	MD, Severe
	12	53.75	f	No	Rumination
	13	54.38	f	–	*(Did not interview)*
	14	55.00	f	–	*(Did not interview)*
	15	55.00	f	No	Mind-wandering
	16	58.13	m	Yes	MD, Mild
	17	59.38	f	Yes	MD, Severe
**D**. ***N*** **=** **127**	1	40.63	f	Yes	MD, Mild
	2	40.63	f	–	*(Did not interview)*
	3	41.25	f	No	Recovered from MD
	4	41.88	f	Yes	MD, Severe
	5	54.38	f	Yes	MD, Severe
	6	57.50	f	No	Immersive Daydreaming, no impairment/distress
	7	57.50	m	–	*(Did not interview)*

*Severity was determined according to the suggested diagnostic criteria and structured clinical interview (3)*.

## Discussion

We found a general rate of 4.2% in the general population (sample A) for self-reported, suspected MD. Although student samples showed higher rates (specifically, 7.6% in sample B, 8.0% in sample C, and a somewhat lower 5.5% in sample D; weighted average across student samples 7.32%), dissecting the community sample according to age groups explained that difference, revealing quite consistent results, with the young adult age group showing the highest rate (8.5% in the 18–30 age group of sample A). Importantly, the interview part of our study revealed that only 60% of those exceeding the self-report cutoff will eventually meet the suggested criteria for a current clinical diagnosis, supporting Hypothesis 1. Taken together, this points to a probable estimated point-prevalence of 2.5% in the general Israeli-Jewish population, and 4.39% in Israeli student samples.

Finding significant current cases of MD in two modest samples of students (*N* = 212 in Sample C, and *N* = 127 in Sample D) suggests that MD is quite prevalent, supporting Hypothesis 2. A prevalence rate of 2.5% is lower than very common disorders such as major depression or specific phobia, and higher than some rarer syndromes like Anorexia Nervosa or even OCD; it is comparable to certain internalizing disorders such as panic disorder (12-months prevalence of 2–3% in adults and adolescents), generalized anxiety disorder (12-months prevalence of 2.9% in adults in the United States), and social anxiety disorder (12-months prevalence of 7% in the United States but 2.3% in Europe), according to the DSM-5 (16). In terms of behavioral addictions, although gambling disorder is quite rare, (16) our resulting prevalence of MD is comparable with gaming disorder, that has provisional status in the DSM-5 but was fully included in the latest revision of the 11th edition of the International Classification of Diseases (ICD-11; https://icd.who.int/en), which has been estimated at 1.96–3.05% (26).

Diagnosed interviewees described the impairing, time-consuming nature of the habit (e.g., “It hurts my ability to focus on a certain task or on the person in front of me, [so] it can sometimes be hurtful or offensive”; “I thought it only takes me a few minutes and recently I started timing it, and I noticed it could go on for an hour and a half”). Interviewees also described the psychological function of MD as an escape from a difficult reality (e.g., “There I live happily ever after, and when I come back to reality and life you can't find yourself. You don't know how to deal with life, with people. There it's fun and I feel good, and in reality it's something else and always brings me down”; “I'm an only child and my mom was at work a lot, so my inner world is very big. It's something that used to save me, otherwise a kid can get lost”; “Sometimes when I'm with people and I get sick of it I feel like going home to daydream”). Several depicted their difficulty in controlling or taming this activity (e.g., “I sometimes want to come out of it and on the other hand I'm in the situation and in [the middle of] something so I don't want to stop”), their shame or sense of secretiveness (e.g., “I wouldn't want people to know this about me”), and their detached experience of existing in two different worlds (e.g., “Almost all the time it haunts me, I feel that it haunts me. It started many years ago. I felt like I don't belong to these people or to this time”).

Hypothesis 3 was partially supported, as MD seemed to be more prevalent among young adults than older adults. In the community sample, when focusing on the age group equivalent to that of the student samples (18–30), suspected prevalence rates rose, reaching rates comparable to those found in the student samples (suggesting that age explained the differences in prevalence between our samples), and even slightly higher. This slightly higher percentage, coupled with the broader range of MDS scores in the community sample, together suggest possibly restricted variance in the student samples, which probably exclude some severe, Non-functional MD cases. A higher prevalence of MD in younger respondents compared to older ones also exists in gaming disorder (26) and attention-deficit hyperactivity disorder (27, 28). Daydreaming may be more prevalent at an age where individuals feel pressure to establish a long-term relationship or when they need to concentrate on studying. Alternatively, cultural cohort factors may play a part (e.g., amount of screen time, exposure to fantasy movies). Despite our hypothesized gender difference (also Hypothesis 3), we did not find a clear pattern that would suggest higher MD prevalence among females. Perhaps the reason for the discrepancy between these results and our encounters in previous studies and personal exchanges stemmed from females being more likely to identify their excessive daydreaming as a problem and look it up online, or perhaps they are simply more likely to volunteer for studies on MD. It is also possible that women and men are different in the type of daydreaming characteristics that trouble them; indeed, previous research indicated differing associations of low life satisfaction and daydreaming, where for women the association was with daydreaming vividness, whereas for men it was with daydreaming frequency (29). Alternatively, the expected gender difference may exist in other countries but not in Israel. Relatedly, prevalence rates of nonfatal suicide attempts are known in the international literature to be much more prevalent in females compared to males (e.g., 76.8% females), (30) but in Israel this gender difference is less obvious (e.g., 57% females in 2016 according to the Israeli ministry of health, based on emergency room data; retrieved July 1st, 2021, https://www.health.gov.il/PublicationsFiles/loss_2018.pdf). Additional studies on MD prevalence in different countries are warranted to further examine this issue.

Finally, Hypothesis 4 was also supported. Most of the MDS-16 items were significantly skewed, specifically items 4–14 and 16, suggesting that MD—as assessed by these items—does not distribute normally in the population, similar to other pathological traits such as dissociation [e.g., (31)]. An average focusing on these specific items may be better suited than all 16 items to screen for clinical-level MD in future studies wishing to conduct clinical interviews. Future studies could focus on these items as screeners by comparing interviews of MD vs. healthy individuals. Regarding the items that seem to be more dimensional (items 1–3 and 15), it is possible that the first three items of the questionnaire have more variance because non-MD respondents do not yet understand the unique nature of immersive daydreaming at this point, whereas only after several items come to realize that these questions do not pertain to them. Notably, item 15 was also the most inconsistently loaded item in an investigation of measurement invariance of the MDS-16 across four countries, (7) suggesting that its meaning is irregular among different people, which may have contributed to its enhanced variance. It is interesting to notice that none of these four items belong to the factor *Impairment*, as that factor was suggested as the best and most consistent indicator of MD, holding the essence to daydreaming as a psychopathology (7). Perhaps impairment items could differentiate well between those suffering from MD and those merely experiencing immersive daydreaming that does not impair functioning. Future studies with larger samples of interviewees could explore this possibility and establish a better screening tool for clinical MD.

This study had several limitations. First, only two samples out of the four samples included clinical interviews. Specifically, our estimate of 2.5% in the general community is based on an approximation (combining the self-reported rates in the community with the rates of diagnosis from the student interviews). Second, not all those who exceeded the cutoff score responded to our invitation to interview, which may have affected the prevalence rates found. On the other hand, the rate of 60% positive MD out of the interviewees was consistent across the two samples; thus, we are quite confident that this figure is representative of a true rate. Third, our results are based on modestly sized, mostly Jew or all Jew, Israeli samples, and do not necessarily generalize to Israeli subcultures (e.g., Arab Israelis) and to other countries. Finally, prevalence in three out of four of the samples in this study may have been affected by the Covid-19 pandemic and its associated restrictions. Indeed, it has been shown that like several other symptoms, MD was also intensified following the outbreak of the pandemic (32). However, comparing the prevalence rates between samples A (4.2%), C (8.0%), and D (5.5%), collected Post-pandemic outbreak, to sample B (7.6%), collected prior to the pandemic outbreak, the pattern does not seem to suggest necessarily higher MD rates in the later samples. It is possible that sample C, the collection of which began during the first lockdown in Israel, may represent such an effect, but the difference between 7.6 and 8.0% does not seem to be large enough to ascertain it. Further research is needed to illuminate context variables that may influence MD prevalence.

To conclude, the present study is the first systematic investigation of MD prevalence in independently recruited (non-MD) samples, including a community sample and three student samples, with structured clinical interviews for those screened in two samples. We found an estimated point-prevalence of 2.5% for MD in the general Israeli population, with higher rates in young adults, and established norms for the distribution of MDS items in normal populations. Future studies should determine norms and prevalence rates in other countries, such as the United States, Europe, and Non-western cultures, as there are many self-identified MDers in numerous countries around the globe [e.g., (15)]. The current study supports the importance of recognizing MD as a specific clinical condition, which affects a small but significant percentage of the population, causing functional impairment, psychological distress, and feelings of shame and inadequateness. Increased recognition of MD by researchers and clinicians may provide respite from loneliness in these individuals and steer them toward mental health.

## Data Availability Statement

The raw data supporting the conclusions of this article will be made available by the authors, without undue reservation.

## Ethics Statement

The studies involving human participants were reviewed and approved by Ben-Gurion University Ethics Review Board. The patients/participants provided their written informed consent to participate in this study.

## Author Contributions

NS-D: writing of the manuscript draft, study concept and design, statistical analysis, interpretation of data, supervision of data collection, and obtained funding. NT-K: data collection, conducting all clinical interviews, and providing critical review for manuscript draft. All authors had full access to all data in the study and take responsibility for the integrity of the data and the accuracy of the data analysis. All authors contributed to the article and approved the submitted version.

## Funding

This research was supported by the Israel Science Foundation Grant No. 539/13 to NS-D. This research was also supported (in part) by the Israel Science Foundation Grant No. 1895/13 to NS-D. The foundation was not involved in the research other than financial support.

## Conflict of Interest

The authors declare that the research was conducted in the absence of any commercial or financial relationships that could be construed as a potential conflict of interest.

## Publisher's Note

All claims expressed in this article are solely those of the authors and do not necessarily represent those of their affiliated organizations, or those of the publisher, the editors and the reviewers. Any product that may be evaluated in this article, or claim that may be made by its manufacturer, is not guaranteed or endorsed by the publisher.

## Footnotes

^1^We terminated recruitment at the start of Operation “Guardian of the Walls” in Israel, as rockets being fired into Israeli cities probably changed stress levels in the population.

^2^ https://fac0c99d-218c-46be-b5c9-06d8b9d5ddbf.usrfiles.com/ugd/fac0c9_0791d1bce773444d8a2ba10d9c2d35f1.pdf

## Abbreviations

MD: Maladaptive Daydreaming
MDers: Maladaptive Daydreamers.
